# Thrombin-induced, TNFR-dependent miR-181c downregulation promotes MLL1 and NF-κB target gene expression in human microglia

**DOI:** 10.1186/s12974-017-0887-5

**Published:** 2017-06-29

**Authors:** Min Yin, Zhiying Chen, Yetong Ouyang, Huiyan Zhang, Zhigang Wan, Han Wang, Wei Wu, Xiaoping Yin

**Affiliations:** 1 Department of Neurology, The Second Affiliated Hospital of Nanchang University, No. 1 Minde Road, Nanchang, 330006 Jiangxi Province China; 2 Department of Neurology, The Affiliated Hospital of Jiujiang University, Jiujiang, 332000 Jiangxi Province China

**Keywords:** Intracerebral hemorrhage, ICH, Thrombin, Microglia, MicroRNA

## Abstract

**Background:**

Controlling thrombin-driven microglial activation may serve as a therapeutic target for intracerebral hemorrhage (ICH). Here, we investigated microRNA (miRNA)-based regulation of thrombin-driven microglial activation using an in vitro thrombin toxicity model applied to primary human microglia.

**Methods:**

A miRNA array identified 22 differential miRNA candidates. Quantitative real-time reverse transcription polymerase chain reaction (qRT-PCR) identified miR-181c as the most significantly downregulated miRNA. TargetScan analysis identified mixed lineage leukemia-1 (MLL1) as a putative gene target for miR-181c. qRT-PCR was applied to assess tumor necrosis factor-alpha (TNF-α), miR-181c, and MLL1 levels following thrombin or proteinase-activated receptor-4-specific activating peptide (PAR4AP) exposure. Anti-TNF-α antibodies and tumor necrosis factor receptor (TNFR) silencing were employed to test TNF-α/TNFR dependence. A dual-luciferase reporter system and miR-181c mimic transfection assessed whether mir-181c directly binds to and negatively regulates MLL1. Nuclear factor kappa-B (NF-κB)-dependent luciferase reporter assays and NF-κB target gene expression were assessed in wild-type (MLL1+) and MLL1-silenced cells.

**Results:**

Thrombin or PAR4AP-induced miR-181c downregulation (*p* < 0.05) and MLL1 upregulation (*p* < 0.05) that were dependent upon TNF-α/TNFR. miR-181c decreased wild-type MLL1 3′-UTR luciferase reporter activity (*p* < 0.05), and a miR-181c mimic suppressed MLL1 expression (*p* < 0.05). Thrombin treatment increased, while miR-181c reduced, NF-κB activity and NF-κB target gene expression in both wild-type (MLL1+) and MLL1-silenced cells (*p* < 0.05).

**Conclusions:**

Thrombin-induced, TNF-α/TNFR-dependent miR-181c downregulation promotes MLL1 expression, increases NF-κB activity, and upregulates NF-κB target gene expression. As miR-181c opposes thrombin’s stimulation of pro-inflammatory NF-κB activity, miR-181c mimic therapy may show promise in controlling thrombin-driven microglial activation following ICH.

**Electronic supplementary material:**

The online version of this article (doi:10.1186/s12974-017-0887-5) contains supplementary material, which is available to authorized users.

## Background

Intracerebral hemorrhage (ICH) displays the most significant mortality and morbidity rates among all stroke subtypes [1]. Unfortunately, there has been limited advancement in the management of ICH beyond conventional measures of controlling blood pressure, thromboprophylaxis, and management of post-ICH complications [2]. Therefore, novel therapeutic approaches based on ICH’s unique pathophysiology are still needed in order to better address the needs of ICH patients.

Specifically, the diffusion of thrombin into the brain parenchyma following ICH has been established as a critical factor in ICH’s neuropathological effects [3]. As a potent extracellular signaling molecule, thrombin produces brain edema, inflammation, and apoptosis through binding and activating thrombin receptors in the central nervous system (CNS) [4]. In addition, thrombin induces secondary brain injury by activating microglia, which subsequently release neurotoxic pro-inflammatory agents—such as tumor necrosis factor-α (TNF-α), interleukin-1α/β (IL-1α/β), interleukin-6 (IL-6), interleukin-12 (IL-12), and cyclooxygenase-2 (COX-2)—into the surrounding brain parenchyma [5, 6]. In particular, thrombin-induced TNF-α release promotes detrimental tumor necrosis factor receptor (TNFR)-dependent microgliotic responses [7–9]. On this basis, controlling thrombin-driven, TNFR-dependent microglial activation may serve as an appropriate therapeutic target for ICH [4].

To this end, modulating the expression of microRNAs (miRNAs)—short non-coding RNAs that post-transcriptionally regulate target mRNAs)—has shown promise as a therapeutic tool for various human CNS disease states [10]. Therefore, in this study, we investigated microRNA-based regulation of thrombin-driven microglial activation using an in vitro thrombin toxicity model applied to primary human microglia. We first determined the differential miRNA profile between thrombin-treated and untreated control microglia. After qRT-PCR validation, we then selected the most significantly downregulated miRNA—hsa-miR-181c (hereinafter miR-181c)—for further investigation. Thereafter, we determined that thrombin-induced miR-181c downregulation is TNF-α/TNFR-dependent and that miR-181c directly binds to and negatively regulates mixed lineage leukemia 1 (MLL1) expression. Finally, in order to better understand the roles of miR-181c and MLL1, we analyzed their effects upon nuclear factor kappa-B (NF-κB) activity and NF-κB target gene expression.

## Methods

### Ethics statement

The Ethics Committee at The Second Affiliated Hospital of Nanchang University (Nanchang, China) approved the protocols of this study. Written informed consent was obtained from all tissue donors prior to inclusion in this study.

### Isolation of human microglia from fresh brain tissue

Adult human microglia were isolated from fresh temporal lobe specimens resected from seven non-demented epileptic patients (four females and three males, mean age [range]; 36.3 years [20–60 years]) at the Department of Neurosurgery at The Second Affiliated Hospital of Nanchang University. In order to isolate the microglia from the brain tissue, Doorn et al.’s optimized protocol for microglia isolation from fresh brain tissue was applied with minor modifications [11]. Briefly, all the following brain tissue protocols were performed on ice. Immediately after resection, the brain tissue specimens (0.4–0.5 g each) were placed in a Petri dish with ice-cold magnesium and calcium-free saline solution/bovine serum albumin (GKN/BSA) buffer (2 ml). The brain tissue specimens were finely minced with two razor blades. The minced brain tissue was then transferred to a 70-μm pore strainer that was placed atop a conical tube. The minced brain tissue was then gently pushed through the strainer to achieve a single-cell suspension in the conical tube. Ice-cold GKN/BSA buffer (30 ml) was then added to the conical tube. The conical tubes were then centrifuged for 10 min at 300 g at 4 °C. The resulting cell pellet was resuspended in 50% Percoll (1 ml). The cell suspension was placed in a 15-ml polystyrene tube, and 50% Percoll (7 ml) was added to the cell suspension. Then, by pipette, 75% Percoll (4 ml) was gently placed beneath the 50% Percoll layer, while GKN/BSA buffer (3 ml) was gently placed on top of the 50% Percoll layer. The cells in the density gradient were then centrifuged for 30 min at 1300 g at 4 °C.

Post-centrifugation, the 50%/75% Percoll interphase contained the isolated microglia. After carefully removing the topmost layer by pipette, the 50%/75% Percoll interphase was aspirated out to a 15-ml polystyrene tube. The cells were rinsed twice with GKN/BSA buffer (14 ml per rinse). After adding GKN/BSA buffer (14 ml), the cells were centrifuged for 7 min at 300 g at 4 °C and resuspended in GKN/BSA buffer (14 ml).

### Magnetic-activated cell sorting (MACS) of human microglia

In order to positively select for microglia and deplete any remaining granulocyte content, Melief et al.’s MACS protocol employing anti-CD15-conjugated magnetic microbeads and anti-CD11b-conjugated magnetic microbeads (Miltenyi Biotec, Bergisch, Gladbach, Germany) was applied as previously described [12]. Briefly, the cells were incubated with anti-CD15-conjugated magnetic microbeads (8 μl microbeads in 100 μl total buffer volume) for 15 min at 4 °C. Cells were then rinsed, resuspended (500 μl buffer), and placed in an MS column within a magnetic field; and the resulting flow-through (consisting of unlabeled cells) was collected. Cells were rinsed, incubated with anti-CD11b-conjugated magnetic microbeads (15 μl microbeads in 100 μl total buffer volume) for 15 min at 4 °C. Cells were rinsed, resuspended (500 μl buffer), and placed in an MS column within a magnetic field. The resulting eluted microglia were then used for culturing.

### Primary culture of human microglia

Microglia were then cultured based on Mulder et al.’s protocol [13]. The microglia were cultured in Dulbecco’s Modified Eagle Medium (DMEM) and HAM-F10 (1:1), 10% fetal calf serum (FCS, *v*/*v*), 2 mM L glutamin, 100 IU/ml penicillin, 50 μg/ml streptomycin, 25 μg/ml granulocyte-monocyte colony stimulating factor (GM-CSF) (all Gibco, Shanghai, China) in a humidified incubator (37 °C, 5% CO_2_). Following 10 days in vitro (DIV), microglia were seeded onto 24-well plates (25000 cells/well) for 24 h of culture before treatment with the in vitro thrombin toxicity model.

### Thrombin chromogenic assay

Thrombin’s proteolytic activity was assessed with S-2238, a chromogenic thrombin substrate (Chromogenix, Sweden) according to Lee et al.’s protocol [14]. Briefly, 100 ml samples of 300 nM thrombin (which equates to 39 U/ml [15]) were pre-incubated in the presence or absence of various concentrations of the thrombin-specific proteolytic inhibitor D-phenylalanyl-L-prolyl-L-arginine chloromethyl ketone dihydrochloride (PPACK, BioMol, Plymouth Meeting, PA, USA) at 37 °C. For a negative control, thrombin (100 ml) was heat-inactivated by boiling (100 °C) for 5 min. After 1 h, 1.5 mM S-2238 (50 ml) was added to the thrombin samples for 4 min. Optical density (OD) was then immediately assessed at 405 nm.

### In vitro thrombin toxicity model

In order to examine thrombin’s effects upon microglia after ICH, an in vitro thrombin toxicity model was constructed by applying pharmaceutical-grade recombinant human thrombin (3490 U/mg; ZymoGenetics, Seattle, WA, USA) to the microglia. Previous in vivo research has established that the acute phase of ICH through day 30 post-ICH produces a peak local thrombin concentration range of ~200–400 nM [16]. In order to best mimic in vivo ICH conditions, we chose 300 nM thrombin (which equates to 39 U/ml [15]) for our in vitro thrombin toxicity model.

Briefly, a new batch of microglia maintained in microglia medium were placed in a chamber with 5% CO_2_ and 100% humidity for 2 days and were then divided into a thrombin group and a control group. The thrombin group was constructed by applying 300 nM thrombin for a period of 24 h to the microglia as previously described [17]. In some experiments, thrombin was replaced with the thrombin receptor agonist proteinase-activated receptor-4 (PAR4)-specific activating peptide (PAR4AP) (300 μM) or another thrombin receptor agonist proteinase-activated receptor-1 (PAR1)-specific activating peptide (PAR1AP) (300 μM). In other experiments, the thrombin-specific proteolytic inhibitor PPACK was added to thrombin to reach the effective PPACK concentration of 5 μM. The control group was left untreated under the same culture conditions. RNA extraction was performed at 1, 3, 6, and 12 h after completion of the 24-h thrombin treatment period. The experiments were performed in triplicate.

### RNA isolation and miRNA array

This procedure was performed as previously described [18]. Total RNA was extracted from the thrombin and control groups using TRIzol reagent (Invitrogen, San Diego, CA, USA) in accordance with manufacturer’s instructions. Small RNAs were isolated using a commercial kit (miRNA Isolation Kit; Ambion, Austin, TX, USA). Array experiments were performed by CapitalBio Corp. (Beijing, China; http://www.capitalbio.com) using a human microRNA (miRNA) array (GeneChip miRNA Array; Affymetrix, Santa Clara, CA, USA), which is composed of probe sets from the miRNAs registered in the Sanger miRBase miRNA database (http://www.mirbase.org). The experiments were performed in triplicate.

### Differential miRNA candidate selection

This procedure was performed as previously described [18]. The miRNA expression profiles of thrombin-treated microglia were compared against control microglia at 3 h post-thrombin treatment by miRNA microarray. The miRNAs that exhibited significantly altered expression levels between the thrombin and control conditions were identified. The miRNAs exhibiting greater than or equal to 1.5-fold downregulation in the thrombin-treated microglia were selected for quantitative real-time reverse transcription polymerase chain reaction (qRT-PCR) validation. The experiments were performed in triplicate.

### qRT-PCR validation of select differential miRNA candidates

This procedure was performed as previously described [18]. qRT-PCR was conducted to compare the relative expression levels (thrombin-treated versus untreated control microglia cells) of the selected downregulated miRNA candidates. TaqMan miRNA assays (Applied Biosystems, Carlsbad, CA, USA) were used to quantify mature miRNA expression levels in accordance with manufacturer’s protocol. For each miRNA candidate, qRT-PCR measurements were performed to obtain a mean CT value for each sample. The CT values of the different samples were compared using the 2^−ΔΔCT^ method with glyceraldehyde-3-phosphate dehydrogenase (GAPDH) expression levels used as an internal reference. The experiments were performed in triplicate.

### Target gene identification

This procedure was performed as previously described [18]. Based on the qRT-PCR findings, miR-181c was found to be the most significantly downregulated microRNA in thrombin-treated microglia cells. Therefore, the TargetScan database (http://www.targetscan.org) was then searched to identify putative human target genes for miR-181c by their 3′ untranslated regions (3′ UTRs). The top five-ranking target genes ranked by TargetScan’s total context score are detailed in Table 1. Based on a thorough review of the relevant literature, the highest-ranking gene—relaxin-like factor (RLF)—showed little association with inflammation or immune response. However, the second-ranking gene—mixed lineage leukemia-1 (MLL1)—showed a strong relevance to inflammation and immune response pathways [19, 20]. Therefore, MLL1 was selected for further investigation as a putative target gene.

**Table 1:** Top five-ranking putative target genes for miR-181c from the TargetScan analysis

Target gene	Representative transcript	Gene name	Total context score	Aggregate *P*_ct_
RLF	NM_012421	Rearranged L-myc fusion	−0.38	0.83
MLL1	NM_001197104	Myeloid/lymphoid or mixed-lineage leukemia 1	−0.37	0.85
ACVR2B	NM_001106	Activin A receptor, type IIB	−0.33	>0.99
XRN1	NM_001042604	5′-3′ Exoribonuclease 1	−0.29	0.69
VHL	NM_000551	Von Hippel-Lindau tumor suppressor	−0.28	0.17

To investigate the relationships between TNF-α, miR-181c, and MLL1 expression, we repeated the in vitro thrombin toxicity model described above. TNF-α, miR-181c, and MLL1 mRNA expression levels at 1, 3, 6, and 12 h after thrombin treatment were measured by qRT-PCR. The experiments were performed in triplicate.

### MiRNA vector construction and luciferase reporter assay for miR-181c and MLL1

This procedure was performed as previously described [18]. In order to validate that miR-181c directly suppresses MLL1 expression, a dual-luciferase reporter system was used. To generate the miRNA expression vector, the miR-181c gene was amplified from human genomic DNA and then cloned into the pcDNA3.0 vector (Invitrogen). The open reading frame (ORF) of human MLL1 was amplified and cloned into the pcDNA3.0 vector.

A MLL1 3′-UTR fragment containing wild-type or mutated miR-181c-binding sites was cloned downstream of the luciferase reporter gene. Mutations were generated in the MLL1 3′-UTR sequences complementary to the seed region of miR-181c. Then, the luciferase complexes were constructed by ligating oligonucleotides containing the wild-type or mutated putative target site of the MLL1 3′ UTR into the multi-cloning site of the p-MIR luciferase reporter vector (Ambion).

A synthetic miRNA mimic duplex corresponding to miR-181c—as well as a negative control (NC) RNA duplex for the miRNA mimic duplex—were designed as described previously [18]. The NC RNA duplex was not homologous to any known human gene sequences. One set of microglia cells was co-transfected with 80 ng of luciferase reporter plasmid, 40 ng of the pRL-TK-Renilla-luciferase plasmid (Promega, Madison, WI, USA), and the miRNA mimic duplex (final concentration of 20 nmol/l). Another set of microglia cells were co-transfected with 80 ng of the luciferase reporter plasmid, 40 ng of the pRL-TK-Renilla-luciferase plasmid (Promega), and the NC RNA duplex (final concentration of 20 nmol/l). The identical co-transfection procedure was performed in HEK293T cells (China Cell Line, Shanghai, China). At 24 h post-transfection, the firefly and Renilla luciferase activities in the microglia and HEK293T cells were measured (Dual-Luciferase Reporter Assay; Promega). The luciferase activity of the NC transfection in each experiment was used to normalize the data; the luciferase activity of the NC transfection was set to unity. Each transfection was repeated twice in triplicate.

### Assessment of MLL1 expression after miR-181c mimic treatment

Microglia cells were transfected with miR-181c mimics or NC. After 48 h, MLL1 mRNA and protein expression were evaluated by qRT-PCR and Western blotting, respectively. Western blotting was conducted according to Zhang et al.’s protocol [18]. Briefly, total protein was extracted, and the total protein content was measured using a commercial bicinchoninic acid (BCA) kit (BCA Protein Assay Kit; Pierce Biotechnology, Rockford, IL, USA). BSA was used as an internal standard. Matching quantities of protein from each group were separated by 10% sodium dodecyl sulfate-polyacrylamide gel electrophoresis (SDS-PAGE) and then transferred to a nitrocellulose membrane (Bio-Rad, Hercules, CA, USA). After blocking with 5% non-fat milk, the membrane was incubated with primary antibodies against human MLL1 (rabbit polyclonal; 1:500 dilution) and human GAPDH (rabbit monoclonal; 1:3000 dilution). The blots were incubated for 2 h with horseradish peroxidase-conjugated secondary antibodies (goat; 1:3000 dilution) in blocking buffer at room temperature. The protein bands were then detected using an enhanced chemiluminescence kit (ECL; Amersham Pharmacia Biotech, Uppsala, Sweden). The experiments were performed in triplicate.

### Reporter assay for NF-κB-dependent luciferase activity

The reporter assay for NF-κB-dependent luciferase activity was conducted as previously described by Lee et al. [21]. Microglia and HEK293T cells were each co-transfected with a NF-κB-responsive luciferase reporter plasmid that contains four κB binding sites (pNF-κB-Luc, 0.5 μg, Clontech) as well as a pSV-β-galactosidase expression plasmid (0.2 μg, Promega). After 24 h, the microglia and HEK293T cells were subjected to various experimental conditions. The resulting luciferase activities were assessed by a luminometer. The findings were normalized to β-galactosidase activity. The experiments were performed in triplicate.

### Knockdown of TNFR and MLL1 expression

Unique siRNAs against human TNFR1, TNFR2, and MLL1 were purchased from Shanghai GenePharma (Shanghai, China) in order to knockdown TNFR and MLL1. For gene silencing, a pSUPER G418 GFP vector containing the relevant siRNA sequence was transfected into the microglia. As a control, a separate population of microglia was transfected with the original pSUPER G418 GFP vector lacking the siRNA sequence. Briefly, forward and reverse oligonucleotide strands containing the relevant siRNA-expressing sequence were annealed. The pSUPER G418 GFP vector was linearized via BgIII and HindIII restriction enzymes. Then, the annealed oligonucleotide strands containing the relevant siRNA-expressing sequence were cloned into the pSUPER GFP G418 vector. The resulting pSUPER GFP G418 vector containing the relevant siRNA-expressing sequence was then transformed into *E. coli*. After culture, the 10 largest *E. coli* colonies were selected. The plasmids extracted from each colony were sequenced to ensure they contained the relevant siRNA-expressing sequence.

One day prior to transfection, microglia were seeded onto six-well plates (500 μl of medium per well) to reach 50% confluence at the moment of transfection. Opti-MEM (60 μl), Superfect transfection reagent (5 μl, Invitrogen), and the cDNA plasmid (2 μg) were mixed and stored for 10 min at room temperature. Microglia were first washed with PBS and washed with Opti-MEM, which was followed by the addition of 350 μl Opti-MEM to each tube. Then, the Opti-MEM was removed and replaced with SuperFect/cDNA. Microglia were then incubated at 37 °C at 5% CO_2_ and 100% humidity. After 3 h of incubation, the medium was changed to microglia medium for an additional culture period of 24 h. Microglia were then screened for the presence of GFP fluorescence through a fluorescence-activated cell sorting (FACS) Aria II. Specifically, the GFP+ microglia subpopulation was sorted apart from the GFP-microglia subpopulation. The GFP+ microglia were cultured for 3 h in serum-free DMEM, and qRT-PCR was then applied to assess mRNA silencing in the GFP+ microglia.

### Assessment of expression of NF-κB target genes

Microglia were transfected with miR-181c mimics or NC. After 48 h, the mRNA expression of four NF-κB target genes—monocyte chemoattractant protein-1 (MCP1), tumor necrosis factor α receptor-associated factor-1 (TRAF1), superoxide dismutase-2 (SOD2), and tumor necrosis factor α-induced protein 3-interacting protein 1 (TNIP1)—was evaluated by qRT-PCR. The experiments were performed in triplicate.

### Statistical analysis

Results were expressed as means ± standard errors of the mean (SEMs) from triplicate experiments where noted. Statistical comparisons between experimental groups were performed using a two-tailed Student’s *t* test or analysis of variance (ANOVA) with a Bonferroni correction. A statistical significance threshold of *p* < 0.05 was applied for all comparisons.

## Results

**Fig. 1:**
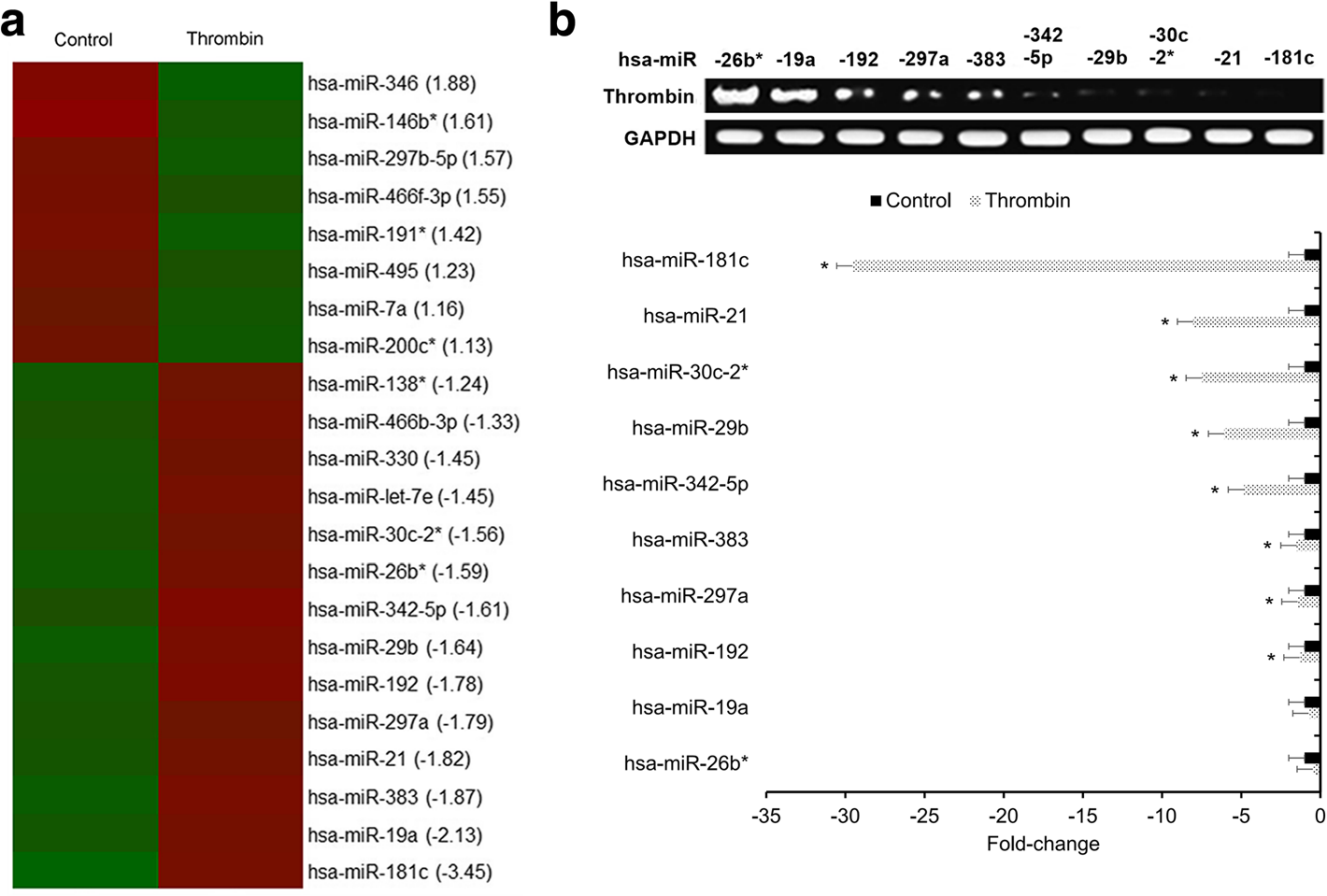
Thrombin downregulates miR-181c in human microglia. **a** Heatmap of the miRNA array identifying differential miRNA candidates. miRNAs exhibiting significantly altered expression between thrombin-treated and untreated control conditions were identified with *red shading* representing downregulation and *green shading* representing upregulation. Experiments performed in triplicate. **b** miRNAs exhibiting ≥1.5 × downregulation in thrombin-treated microglia were selected for qRT-PCR validation. For each miRNA candidate, qRT-PCR measurements were performed to obtain a mean CT value for each sample. CT values of the different samples were compared using the 2^−ΔΔCT^ method with GAPDH expression levels used as an internal reference. All results presented as means ± SEMs from three independent experiments. *p < 0.05, downregulation versus control

An Affymetrix GeneChip miRNA Array was used to identify differential miRNA candidates in primary human microglia under thrombin-treated versus control conditions. A total of 22 miRNAs exhibited significantly altered expression levels between thrombin-treated and untreated control microglia (Fig. 1a). Only the miRNAs exhibiting greater than or equal to a 1.5-fold downregulation in thrombin-treated microglia were selected for qRT-PCR validation. As a result, miR-181c was found to be the most significantly downregulated miRNA in thrombin-treated microglia relative to untreated control microglia (Fig. 1b) and was selected for further investigation.

TargetScan analysis was then applied to identify putative gene targets for miR-181c. MLL1 was the highest-ranking target gene that showed a strong relevance to inflammation and immune response (Table 1), and further, TargetScan analysis revealed four putative binding sites for miR-181c on the 3′ UTR of MLL1 (Additional file 1: Figure S1). Therefore, MLL1 was selected for further investigation as a candidate target gene of miR-181c.

**Fig. 2:**
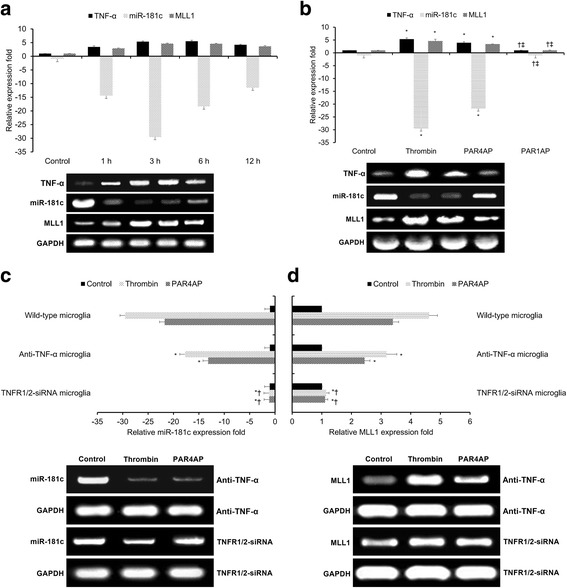
Thrombin-driven, PAR4-mediated TNF-α expression downregulates miR-181c and upregulates MLL1. **a** qRT-PCR comparing relative expression levels of TNF-α, miR-181c, and MLL1 at 1, 3, 6, and 12 h after 24-h thrombin treatment. Control indicates untreated control microglia. **b** qRT-PCR comparing relative expression levels of TNF-α, miR-181c, and MLL1 at 3 h following treatment with thrombin, PAR4AP, or PAR1AP. *p < 0.05 versus control, †*p* < 0.05 versus thrombin, ‡*p* < 0.05 versus PAR4AP. **c**, **d** qRT-PCR comparing relative expression levels of **c** miR-181c and **d** MLL1 at 3 h following treatment with thrombin or PAR4AP after blocking TNF-α or silencing TNFR1/2. *p < 0.05 versus wild-type microglia, †*p* < 0.05 versus anti-TNF-α microglia. All results presented as means ± SEMs from three independent experiments. *p < 0.05 versus control

We then investigated the relationship(s) between miR-181c, MLL1, and the key thrombin-induced pro-inflammatory mediator TNF-α in response to thrombin [22]. Both TNF-α as well as MLL1 were found to be upregulated following thrombin treatment (Fig. 2a), and we confirmed inverse relationships between TNF-α and MLL1 expression versus miR-181c levels at 1, 3, 6, and 12 h post-thrombin treatment (Fig. 2a). Moreover, treatment with the thrombin receptor agonist PAR4AP induced effects similar to thrombin on TNF-α, miR-181c, and MLL1 expression (Fig. 2b), while treatment with another thrombin receptor agonist PAR1AP failed to induce similar effects to thrombin (Fig. 2b). These combined findings suggest that thrombin-driven PAR4 activity in human microglia promotes TNF-α and MLL1 expression while suppressing miR-181c levels.

In order to further investigate the relationship(s) between TNF-α, miR-181c, and MLL1 in response to thrombin, we applied TNF-α blocking antibodies, TNFR1/2 silencing, and the thrombin-specific proteolytic inhibitor PPACK to thrombin-treated human microglia. Both anti-TNF-α antibodies and TNFR1/2 silencing significantly suppressed thrombin’s (and PAR4AP’s) effects upon miR-181c levels (Fig. 2c) and MLL1 expression (Fig. 2d), with TNFR1/2 silencing having a more pronounced effect than anti-TNF-α antibodies. Moreover, the addition of the thrombin-specific proteolytic inhibitor PPACK significantly inhibited thrombin’s effects upon miR-181c or MLL1 expression (Additional file 2: Figure S2). These combined findings suggest that thrombin-driven PAR4 activity in human microglia promotes MLL1 expression and suppresses miR-181c levels via TNF-α/TNFR.

**Fig. 3:**
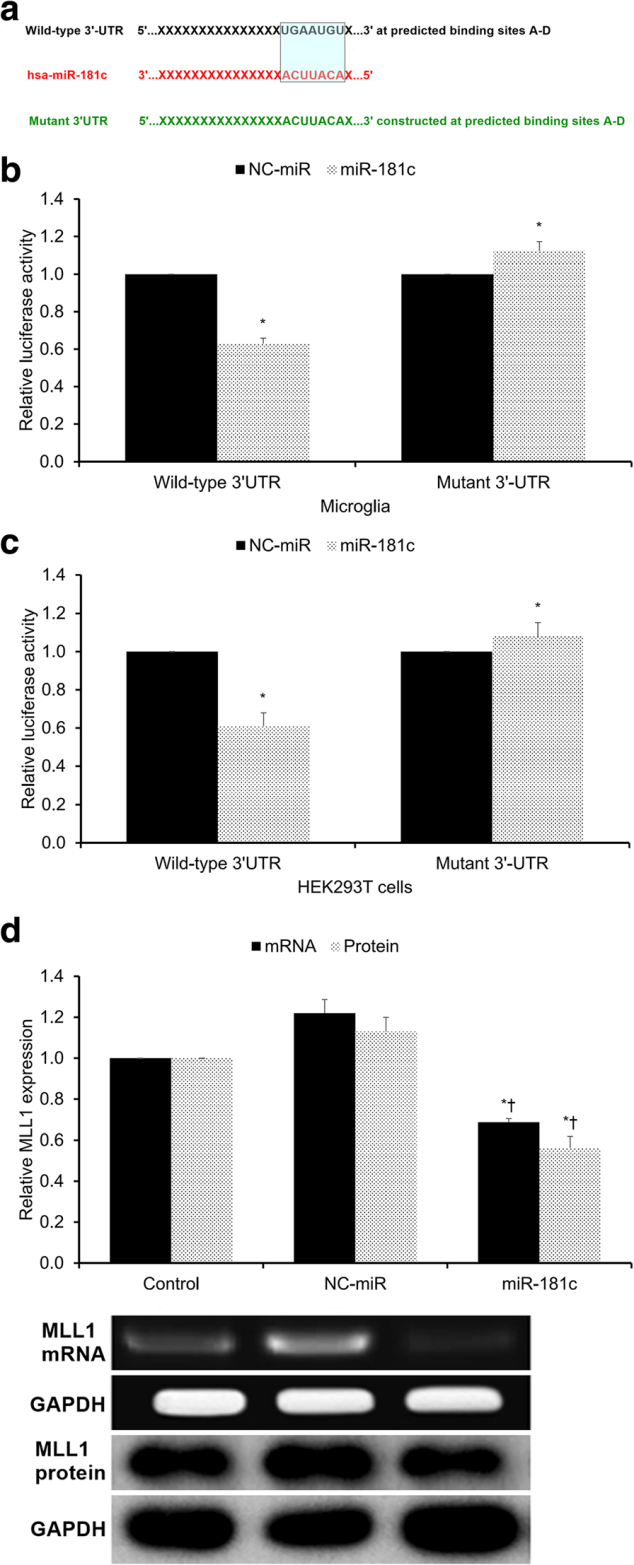
miR-181c suppresses MLL1 expression via 3′-UTR binding. **a** Human MLL1 3′-UTR fragments containing either wild-type or mutated miR-181c-binding sites cloned downstream of the luciferase reporter gene. Mutations were generated in MLL1’s 3′-UTR sequences complementary to miR-181c’s seed region. **b**, **c** Luciferase activity assays using reporters with either wild-type or mutant human MLL1’s 3′-UTRs performed following co-transfection with miR-181c mimics or NC-miR in **b** microglia or **c** HEK293T cells. Luciferase activity of the NC-miR transfection (which was set to unity) used for normalization. **d** Microglia transfected with miR-181c mimic or NC-miR duplexes. After 48 h, MLL1’s mRNA and protein expression were evaluated by qRT-PCR and Western blotting, respectively. Control indicates untreated control microglia. NC-miR indicates the negative control for the miR-181c mimics. All results presented as means ± SEMs from three independent experiments. *p < 0.05 versus same-condition control, †*p* < 0.05 versus same-condition NC-miR

As MLL1 and miR-181c levels show an inverse relationship and TargetScan analysis revealed four putative miR-181c binding sites on MLL1’s 3′ UTR (Additional file 1: Figure S1), we hypothesized that miR-181c may directly regulate MLL1 expression in human microglia. In order to test this hypothesis, a dual-luciferase reporter system was used to assess whether mir-181c directly binds to and regulates MLL1. Human MLL1 3′-UTR fragments containing either wild-type or mutated miR-181c-binding sites were cloned downstream of the luciferase reporter gene (Fig. 3a). We found that miR-181c significantly decreased wild-type (but not mutant) 3′-UTR luciferase reporter activity in both microglia (*p* < 0.05, Fig. 3b) and HEK293T cells (*p* < 0.05, Fig. 3c), indicating that miR-181c directly suppresses MLL1 expression through binding to its wild-type 3′ UTR. Then, microglia were transfected with either miR-181c mimic or NC duplexes. The miR-181c mimic significantly suppressed both MLL1 mRNA and protein expression (*p* < 0.05, Fig. 3d). In sum, the foregoing findings reveal that miR-181c negatively regulates MLL1 expression by binding to its wild-type 3′ UTR.

**Fig. 4:**
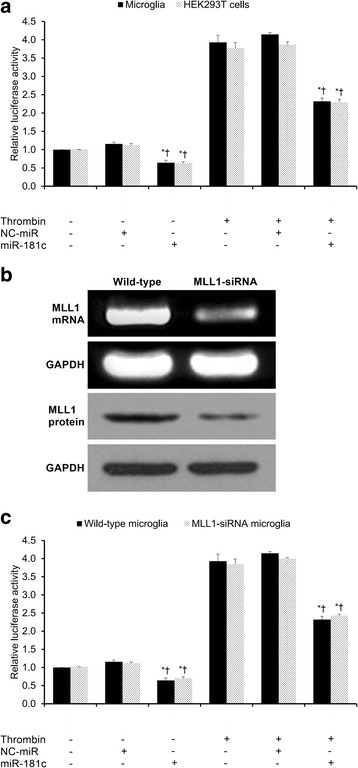
Assessment of NF-κB activity by luciferase assay. **a** Relative NF-κB luciferase activity in microglia and HEK293T cells under the various experimental conditions. **b** MLL1 knockdown in MLL1-silenced microglia was validated by qRT-PCR and Western blotting. **c** Relative NF-κB luciferase activity in MLL1+ and MLL1-silenced microglia under the various experimental conditions. Control condition indicates untreated cells, while thrombin indicates thrombin-treated cells. NC-miR indicates the negative control for the miR-181c mimics. All results presented as means ± SEMs from three independent experiments. *p < 0.05 versus same-condition control, †*p* < 0.05 versus same-condition NC-miR, ‡*p* < 0.05, same-condition wild-type versus MLL1-siRNA microglia

As thrombin activates the transcription factor NF-κB and MLL1 is recruited by NF-κB’s p52 subunit, we tested NF-κB activity through a NF-κB-dependent luciferase reporter assay under various experimental conditions. Thrombin treatment significantly increased NF-κB activity in both microglia and HEK293T cells (*p* < 0.05, Fig. 4a). Under both the untreated control and thrombin-treated conditions, miR-181c significantly reduced NF-κB activity in both microglia and HEK293T cells (*p* < 0.05, Fig. 4a).

We then repeated the identical experimental procedure in order to compare NF-κB activity in wild-type (MLL1+) and MLL1-silenced microglia, wherein MLL1 knockdown was validated by qRT-PCR and Western blotting (Fig. 4b). Thrombin treatment significantly increased NF-κB activity in both wild-type and MLL1-silenced microglia (*p* < 0.05, Fig. 4c). Moreover, under both the untreated control and thrombin-treated conditions, miR-181c significantly reduced NF-κB activity in both wild-type and MLL1-silenced microglia (*p* < 0.05, Fig. 4c). Under all experimental conditions, there were no significant differences in NF-κB activity detected between wild-type and MLL1-silenced microglia (*p* > 0.05, Fig. 4c). In sum, under both untreated control and thrombin-treated conditions, MLL1 expression has a negligible effect upon NF-κB activity, while miR-181c significantly reduces NF-κB activity independent of MLL1.

**Fig. 5:**
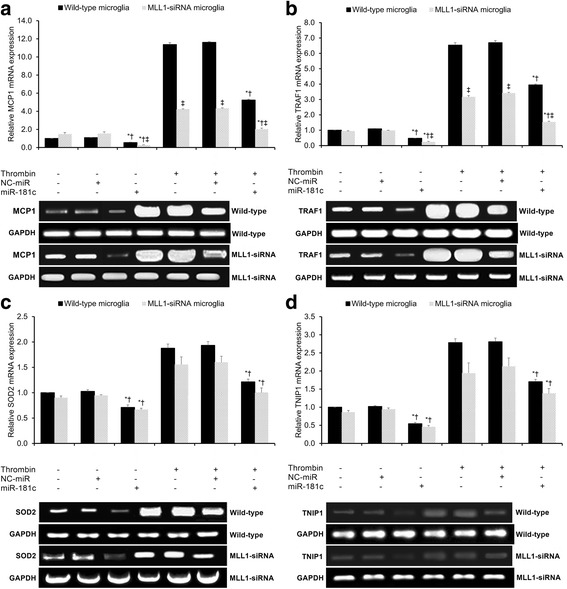
Assessment of NF-κB target gene expression by qRT-PCR. qRT-PCR assessing relative mRNA expression of **a** MCP1, **b** TRAF1, **c** SOD2, and **d** TNIP1 under the various experimental conditions. Control condition indicates untreated cells, while thrombin indicates thrombin-treated cells. NC-miR indicates the negative control for the miR-181c mimics. All results presented as means ± SEMs from three independent experiments. *p < 0.05 versus same-condition control, †*p* < 0.05 versus same-condition NC-miR, ‡*p* < 0.05, same-condition wild-type versus MLL1-siRNA microglia

We then studied the role of MLL1 in NF-κB signaling by comparatively assaying the mRNA expression of NF-κB target genes in wild-type and MLL1-silenced microglia. Four NF-κB target genes were assayed: two genes which have previously demonstrated MLL1 dependence (MCP1 and TRAF1) and two genes which have previously demonstrated MLL1 independence (SOD2 and TNIP1) [19]. Thrombin treatment significantly increased MCP1 expression in both wild-type and MLL1-silenced microglia (*p* < 0.05, Fig. 5a). Under both the untreated control and thrombin-treated conditions, miR-181c significantly reduced MCP1 expression in both wild-type and MLL1-silenced microglia (*p* < 0.05, Fig. 5a). Under all experimental conditions, MLL1-silenced microglia showed significantly lower MCP1 expression relative to wild-type microglia (*p* < 0.05, Fig. 5a). The same pattern of findings was found for TRAF1 (Fig. 5b). In sum, under both untreated control and thrombin-treated conditions, MLL1 expression has a significantly positive effect upon MCP1 and TRAF1 expression, while miR-181c significantly reduces MCP1 and TRAF1 expression independent of MLL1.

We then repeated the identical experimental procedure in order to compare SOD2 and TNIP1 mRNA expression in wild-type and MLL1-silenced microglia. Thrombin treatment significantly increased SOD2 expression in both wild-type and MLL1-silenced microglia (*p* < 0.05, Fig. 5c). Under both the untreated control and thrombin-treated conditions, miR-181c significantly reduced SOD2 expression in both wild-type and MLL1-silenced microglia (*p* < 0.05, Fig. 5c). Under all experimental conditions, there was no significant difference in SOD2 expression between wild-type and MLL1-silenced microglia (*p* > 0.05, Fig. 5c). The same pattern of findings was found for TNIP1 (Fig. 5d). In sum, under both untreated control and thrombin-treated conditions, MLL1 expression has negligible effects upon SOD2 and TNIP1 expression, while miR-181c significantly reduced SOD2 and TNIP1 expression independent of MLL1.

## Discussion

In this study, we investigated microRNA-based regulation of thrombin-driven microglial activation in primary human microglia using an in vitro thrombin toxicity model. First, we found that thrombin or PAR4AP induces significant miR-181c downregulation and significant MLL1 upregulation in primary human microglia that are dependent upon TNF-α/TNFR. Second, we found that miR-181c directly binds to and negatively regulates MLL1 expression in microglia. Third, we found that miR-181c was able to negatively regulate NF-κB activity independent of MLL1. Fourth, while MLL1 expression significantly impacts MLL1-dependent NF-κB target gene expression (i.e., MCP1 and TRAF1) but showed negligible impact upon MLL1-independent NF-κB target gene expression (i.e., SOD2 and TNIP1), miR-181c was able to significantly reduce the expression of all four NF-κB target genes independent of MLL1. In sum, thrombin-induced, TNF-α/TNFR-dependent miR-181c downregulation promotes MLL1 expression, increases NF-κB activity, and upregulates NF-κB target gene expression in human microglia (Additional file 3: Figure S3).

The microRNA miR-181c has been previously linked with several neurological pathologies, including lipopolysaccharide (LPS)-induced miR-181c downregulation in mice [23], miR-181c downregulation in amyloid β-depositing APP23 transgenic mice and human Alzheimer’s disease tissue [24], middle cerebral artery occlusion-induced miR-181c upregulation in rats [25], and differential miR-181c expression in cerebrospinal fluid samples from multiple sclerosis patients relative to those from other neurologic diseases [26]. With specific respect to microglia cells, Zhang et al. discovered that oxygen-glucose deprivation (OGD) significantly downregulates miR-181c expression in rodent microglial cells [18, 27]. Consistent with Zhang et al.’s findings, we found that thrombin also induced significant miR-181c downregulation in human microglia (Fig. 1b).

Moreover, consistent with Hutchinson et al.’s work in cultured astrocytes [23], we also showed that this thrombin-induced miR-181c downregulation is dependent upon TNF-α/TNFR (Fig. 2). Specifically, treatment with thrombin or PAR4AP was able to induce TNF-α expression and miR-181c downregulation, an effect reversed by anti-TNF-α antibody therapy or TNFR1/2 silencing. Of note, several previous studies in microglia have provided conflicting results regarding thrombin’s effect upon TNF-α expression. Early studies in this field were plagued by the use of contaminated plasma-derived thrombin as opposed to pharmaceutical-grade recombinant thrombin [28]. However, even the proper use of recombinant thrombin has produced conflicting results regarding TNF-α release in microglia. For example, consistent with our findings, Suo et al. demonstrated that recombinant thrombin and PAR4AP (but not PAR1AP) induced dose-dependent TNF-α release in murine microglia in vitro [22, 29]. In contrast, Weinstein et al. found that recombinant thrombin failed to trigger TNF-α expression in murine microglia in vitro [15]. As these two previous studies used different thrombin suppliers and different culture conditions, it is possible that these discrepancies in TNF-α release may be attributable to differences in the recombinant thrombin stocks or culture conditions. Moreover, as these two previous studies used murine microglia, our findings may also be attributable to inherent differences in PAR4 expression and/or sensitivity between cultured human and murine microglia. Further research is required to elucidate thrombin’s role in TNF-α release from human microglia.

The transcription factor NF-κB is well regarded as a key mediator of inflammation [30]. Dysregulated NF-κB activation has been linked with various inflammatory disease states, including Alzheimer’s disease, arthritis, asthma, atherosclerosis, chronic obstructive pulmonary disease, idiopathic pulmonary fibrosis, and inflammatory bowel disease [30]. NF-κB is a dimer that can be composed of either a RELA (p65)/p50 complex (NFKB1, which participates in the canonical pathway) or a RELB/p52 complex (NFKB2, which participates in the non-canonical pathway) [31]. The canonical pathway primarily affects inflammation, cellular survival, and cellular proliferation, while the non-canonical pathway primarily affects lymphomagenesis [31]. With respect to the canonical pathway, several pro-inflammatory factors—including thrombin, TNF-α, IL-1, and LPS—have been shown to activate the RELA (p65)/p50 complex through phosphorylating IκB via the IκB kinase (IKK) complex, an action which allows nuclear translocation of the RELA (p65)/p50 complex and subsequent target gene expression [30, 31]. Consistent with these previous findings, we confirmed that thrombin treatment significantly increases NF-κB activity in both microglia and HEK293T cells (Fig. 4a). In addition, consistent with Zhang et al.’s findings in rat microglial cells [27], we found that miR-181c also inhibited NF-κB activation in human microglia (Fig. 4a).

MLL1 is an H3K4 methyltransferase possessing a conserved catalytic SET domain for methylating histone H3K4 [32]. MLL1 functions as a key transcription factor, regulating ~5% of actively transcribed genes [33]. Although the precise mechanism by which MLL1 regulates gene transcription remains unknown, MLL1 typically associates with transcriptionally active chromatins in G1 phase and dissociates from condensed chromatin during mitosis [34]. With regard to the relationship between NF-κB and MLL1, Robert et al. demonstrated that p52 recruits MLL1 to the matrix metalloproteinase-9 promoter [35], Kuo et al. showed that the RELA (p65)/p50 complex is required for MLL1’s attachment to the promoters of its target genes [36], and Wang et al. demonstrated that MLL1 associates with RELA (p65) in the cytoplasm and promotes the expression of NF-κB target genes in TNF-α and LPS models without altering RELA activity [19]. Consistent with Wang et al.’s findings, we confirmed that NF-κB activity was not significantly altered by MLL1 silencing in microglia (Fig. 4c). Moreover, although we showed that miR-181c binds to and negatively regulates MLL1 expression (Fig. 3), we found that miR-181c inhibited NF-κB activation in microglia independent of MLL1 (Fig. 4c).

Having established the effects of miR-181c and MLL1 upon NF-κB activation, we next analyzed their effects upon NF-κB target genes. Wang et al. identified a subset of MLL1-dependent genes whose expression was significantly impaired through MLL1 silencing (e.g., MCP1, TRAF1, NF-κ light chain gene enhancer in B cells inhibitor alpha (NFKBIA), and A20) and a subset of MLL1-independent genes whose expression was not significantly altered through MLL1 silencing (e.g., SOD2 and TNIP1), suggesting that MLL1 selectively regulates NF-κB target genes [19]. Therefore, here, we selected two MLL1-dependent genes (MCP1 and TRAF1) and two MLL1-independent genes (SOD2 and TNIP1) in order to test this hypothesis in microglia. We found that MLL1 expression significantly impacts MLL1-dependent NF-κB target gene expression (i.e., MCP1 and TRAF1; Fig. 5a, b) but showed negligible impact upon MLL1-independent NF-κB target gene expression (i.e., SOD2 and TNIP1; Fig. 5c, d). However, miR-181c significantly reduced the expression of all four of these NF-κB target genes independent of MLL1 (Fig. 5).

These combined findings suggest that, although miR-181c negatively regulates MLL1 expression, miR-181c is able to inhibit NF-κB activation and downregulate NF-κB target gene expression through mechanism(s) independent of MLL1. Several previous studies have shown that miR-181c negatively regulates other key signaling proteins (such as TNF-α) that are able to activate NF-κB and promote expression of its target genes [18]. Therefore, we hypothesize that miR-181c acts to suppress NF-κB activation and downregulate NF-κB target gene expression through these other NF-κB-associated signaling pathways. As NF-κB activation has been shown to promote the expression of various pro-inflammatory factors (e.g., TNF-α, IL-1β, and inducible nitric oxide synthase (iNOS)) [27] and we have shown that miR-181c suppresses NF-κB activation in human microglia under thrombin-treated conditions, the current findings suggest that miR-181c mimic therapy may show promise in controlling thrombin-driven microglial activation following ICH.

There are several limitations to this study. First, these findings concern an in vitro thrombin toxicity model of primary human microglia cultured from resected brain tissue, which may not necessarily reflect the post-ICH in vivo changes to human microglia nor those for other human brain cell types. Second, although this study suggests that thrombin-driven PAR4 activity induces significant miR-181c downregulation that is dependent upon TNF-α/TNFR, the precise mechanism by which the TNF-α/TNFR pathway downregulates miR-181c in microglia remains an open question. Third, although we show that miR-181c is capable of negatively regulating NF-κB activity and the expression of NF-κB target genes independent of MLL1, the precise alternative mechanism(s) by which it does so remain unclear.

## Conclusions

In conclusion, thrombin-induced, TNF-α/TNFR-dependent miR-181c downregulation promotes MLL1 expression, increases NF-κB activity, and upregulates NF-κB target gene expression in human microglia. As miR-181c opposes thrombin’s stimulation of pro-inflammatory NF-κB activity, these findings suggest that miR-181c mimic therapy may show promise in controlling thrombin-driven microglial activation following ICH. Further study on the molecular effects of miR-181c across a broader range of brain cells is needed to assess any potential therapeutic benefits for ICH.

## Additional files

Additional file 1: Figure S1. miR-181c’s putative binding sites on MLL1’s 3′-UTR. Four putative binding sites for miR-181c on the 3′-UTR of MLL1 based on the TargetScan search. (TIF 429 kb)

Additional file 2: Figure S2. Thrombin’s proteolytic activity contributed to its effects upon miR-181c and MLL1 expression in human microglia. (A) Validation of the thrombin-specific proteolytic inhibitor PPACK’s inhibition of thrombin activity. Thrombin’s proteolytic activity was measured via a chromogenic assay following pre-incubation in the absence or presence of various concentrations of PPACK. Heat-inactivated (boiled) thrombin was applied as a negative control. *p < 0.05 versus control, †*p* < 0.05 versus boiled thrombin, ‡*p* < 0.05 versus thrombin. (B) Pre-incubating with PPACK significantly inhibited thrombin’s effects upon miR-181c and MLL1 expression. *p < 0.05 versus control, †*p* < 0.05 versus thrombin. (TIF 598 kb)

Additional file 3: Figure S3. Schematic overview of thrombin’s effects upon miR-181c and MLL1 in human microglia. Thrombin (via PAR4) induces TNF-α secretion from human microglia [21]. Thrombin-induced TNF-α (via TNFR) suppresses miR-181c levels. This suppression of the inhibitory miR-181c promotes MLL1 expression, increases NF-κB activity, and upregulates downstream NF-κB target gene expression in human microglia. (JPG 456 kb)

## Abbreviations

3′ UTR: 3′ Untranslated region
ANOVA: Analysis of variance
BCA: Bicinchoninic acid
CNS: Central nervous system
COX-2: Cyclooxygenase-2
DIV: Days in vitro
DMEM: Dulbecco’s Modified Eagle Medium
FACS: Fluorescence-activated cell sorting
FCS: Fetal calf serum
GAPDH: Glyceraldehyde-3-phosphate dehydrogenase
GKN/BSA: Magnesium and calcium free saline solution/bovine serum albumin
GM-CSF: Granulocyte-monocyte colony stimulating factor
ICH: Intracerebral hemorrhage
IKK: IκB kinase
IL-12: Interleukin-12
IL-1α/β: Interleukin-1α/β
IL-6: Interleukin-6
iNOS: Inducible nitric oxide synthase
LPS: Lipopolysaccharide
MACS: Magnetic-activated cell sorting
MCP1: Monocyte chemoattractant protein-1
miRNA: MicroRNA
MLL1: Mixed lineage leukemia-1
NC: Negative control
NFKBIA: NF-κ light chain gene enhancer in B cells inhibitor alpha
NF-κB: Nuclear factor kappa-B
OGD: Oxygen-glucose deprivation
ORF: Open reading frame
PAR1: Proteinase-activated receptor-1
PAR1AP: PAR1-specific activating peptide
PAR4: Proteinase-activated receptor-4
PAR4AP: PAR4-specific activating peptide
PPACK: D-phenylalanyl-L-prolyl-L-arginine chloromethyl ketone dihydrochloride
qRT-PCR: Quantitative real-time reverse transcription polymerase chain reaction
RLF: Relaxin-like factor
SDS-PAGE: Sodium dodecyl sulfate-polyacrylamide gel electrophoresis
SEMs: Standard errors of the mean
SOD2: Superoxide dismutase-2
TNFR: Tumor necrosis factor receptor
TNF-α: Tumor necrosis factor-α
TNIP1: Tumor necrosis factor α-induced protein-3-interacting protein 1
TRAF1: Tumor necrosis factor α receptor-associated factor-1

## References

[1] Jones SB, Sen S, Lakshminarayan K, Rosamond WD (2013). Poststroke outcomes vary by pathogenic stroke subtype in the Atherosclerosis Risk in Communities Study. Stroke.

[2] Hemphill JC, Greenberg SM, Anderson CS, Becker K, Bendok BR, Cushman M, Fung GL, Goldstein JN, Macdonald RL, Mitchell PH (2015). Guidelines for the management of spontaneous intracerebral hemorrhage a guideline for healthcare professionals from the american heart association/american stroke association. Stroke.

[3] Liu C, Shi B, Zhou J (2014). Effects of thrombin on the secondary cerebral injury of perihematomal tissues of rats after intracerebral hemorrhage. Genet Mol Res.

[4] Babu R, Bagley JH, Di C, Friedman AH, Adamson C (2012). Thrombin and hemin as central factors in the mechanisms of intracerebral hemorrhage–induced secondary brain injury and as potential targets for intervention. Neurosurg Focus.

[5] Davalos D, Baeten KM, Whitney MA, Mullins ES, Friedman B, Olson ES, Ryu JK, Smirnoff DS, Petersen MA, Bedard C (2014). Early detection of thrombin activity in neuroinflammatory disease. Ann Neurol.

[6] Krenzlin H, Lorenz V, Danckwardt S, Kempski O, Alessandri B (2016). The importance of thrombin in cerebral injury and disease. Int J Mol Sci.

[7] Hua Y, Wu J, Keep RF, Nakamura T, Hoff JT, Xi G (2006). Tumor necrosis factor-α increases in the brain after intracerebral hemorrhage and thrombin stimulation. Neurosurgery.

[8] Cui G, Zuo T, Zhao Q, Hu J, Jin P, Zhao H, Jing J, Zhu J, Chen H, Liu B (2013). ROCK mediates the inflammatory response in thrombin induced microglia. Neurosci Lett.

[9] King MD, Alleyne CH, Dhandapani KM (2013). TNF-alpha receptor antagonist, R-7050, improves neurological outcomes following intracerebral hemorrhage in mice. Neurosci Lett.

[10] Madathil SK, Nelson PT, Saatman KE, Wilfred BR (2011). MicroRNAs in CNS injury: potential roles and therapeutic implications. Bioessays.

[11] Doorn KJ, Brevé JJ, Drukarch B, Boddeke HW, Huitinga I, Lucassen PJ, van Dam A-M (2015). Brain region-specific gene expression profiles in freshly isolated rat microglia. Front Cell Neurosci.

[12] Melief J, Koning N, Schuurman KG, Van De Garde MD, Smolders J, Hoek RM, Van Eijk M, Hamann J, Huitinga I (2012). Phenotyping primary human microglia: tight regulation of LPS responsiveness. Glia.

[13] Mulder SD, Nielsen HM, Blankenstein MA, Eikelenboom P, Veerhuis R (2014). Apolipoproteins E and J interfere with amyloid-beta uptake by primary human astrocytes and microglia in vitro. Glia.

[14] Lee DY, Oh YJ, Jin BK (2005). Thrombin-activated microglia contribute to death of dopaminergic neurons in rat mesencephalic cultures: dual roles of mitogen-activated protein kinase signaling pathways. Glia.

[15] Weinstein JR, Hong S, Kulman JD, Bishop C, Kuniyoshi J, Andersen H, Ransom BR, Hanisch UK, Möller T (2005). Unraveling thrombin’s true microglia-activating potential: markedly disparate profiles of pharmaceutical-grade and commercial-grade thrombin preparations. J Neurochem.

[16] Rooth E (2011). Hemostatic disturbances in acute ischemic stroke.

[17] Kim J-M, Lee S-T, Chu K, Jung K-H, Kim JH, Yu J-S, Kim S, Kim SH, Park D-K, Moon J (2014). Inhibition of Let7c MicroRNA is neuroprotective in a Rat intracerebral hemorrhage model.

[18] Zhang L, Dong L-Y, Li Y-J, Hong Z, Wei W-S (2012). The microRNA miR-181c controls microglia-mediated neuronal apoptosis by suppressing tumor necrosis factor. J Neuroinflammation.

[19] Wang X, Zhu K, Li S, Liao Y, Du R, Zhang X, Shu H-B, Guo A-Y, Li L, Wu M (2012). MLL1, a H3K4 methyltransferase, regulates the TNFα-stimulated activation of genes downstream of NF-κB. J Cell Sci.

[20] Yamashita M, Hirahara K, Shinnakasu R, Hosokawa H, Norikane S, Kimura MY, Hasegawa A, Nakayama T (2006). Crucial role of MLL for the maintenance of memory T helper type 2 cell responses. Immunity.

[21] Lee JY, Jhun BS, Oh YT, Lee JH, Choe W, Baik HH, Ha J, Yoon K-S, Kim SS, Kang I (2006). Activation of adenosine A 3 receptor suppresses lipopolysaccharide-induced TNF-α production through inhibition of PI 3-kinase/Akt and NF-κB activation in murine BV2 microglial cells. Neurosci Lett.

[22] Suo Z, Wu M, Citron BA, Gao C, Festoff BW (2003). Persistent protease-activated receptor 4 signaling mediates thrombin-induced microglial activation. J Biol Chem.

[23] Hutchison ER, Kawamoto EM, Taub DD, Lal A, Abdelmohsen K, Zhang Y, Wood WH, Lehrmann E, Camandola S, Becker KG (2013). Evidence for miR-181 involvement in neuroinflammatory responses of astrocytes. Glia.

[24] Schonrock N, Humphreys DT, Preiss T, Götz J (2012). Target gene repression mediated by miRNAs miR-181c and miR-9 both of which are down-regulated by amyloid-β. J Mol Neurosci.

[25] Jeyaseelan K, Lim KY, Armugam A (2008). MicroRNA expression in the blood and brain of rats subjected to transient focal ischemia by middle cerebral artery occlusion. Stroke.

[26] Haghikia A, Haghikia A, Hellwig K, Baraniskin A, Holzmann A, Décard BF, Thum T, Gold R (2012). Regulated microRNAs in the CSF of patients with multiple sclerosis: a case-control study. Neurology.

[27] Zhang L, Li YJ, Wu XY, Hong Z, Wei WS (2015). MicroRNA-181c negatively regulates the inflammatory response in oxygen-glucose-deprived microglia by targeting Toll-like receptor 4. J Neurochem.

[28] Möller T, Weinstein JR, Hanisch U-K (2006). Activation of microglial cells by thrombin: past, present, and future. Seminars in thrombosis and hemostasis.

[29] Suo Z, Wu M, Ameenuddin S, Anderson HE, Zoloty JE, Citron BA, Andrade‐Gordon P, Festoff BW (2002). Participation of protease-activated receptor-1 in thrombin-induced microglial activation. J Neurochem.

[30] Rahman A, Fazal F (2011). Blocking NF-κB: an inflammatory issue. Proc Am Thorac Soc.

[31] Goyama S, Mulloy JC (2013). NF-κB: a coordinator for epigenetic regulation by MLL. Cancer Cell.

[32] Dou Y, Milne TA, Ruthenburg AJ, Lee S, Lee JW, Verdine GL, Allis CD, Roeder RG (2006). Regulation of MLL1 H3K4 methyltransferase activity by its core components. Nat Struct Mol Biol.

[33] Wang P, Lin C, Smith ER, Guo H, Sanderson BW, Wu M, Gogol M, Alexander T, Seidel C, Wiedemann LM (2009). Global analysis of H3K4 methylation defines MLL family member targets and points to a role for MLL1-mediated H3K4 methylation in the regulation of transcriptional initiation by RNA polymerase II. Mol Cell Biol.

[34] Mishra BP, Ansari KI, Mandal SS (2009). Dynamic association of MLL1, H3K4 trimethylation with chromatin and Hox gene expression during the cell cycle. Febs Journal.

[35] Robert I, Aussems M, Keutgens A, Zhang X, Hennuy B, Viatour P, Vanstraelen G, Merville M-P, Chapelle J-P, de Leval L (2009). Matrix metalloproteinase-9 gene induction by a truncated oncogenic NF-κB2 protein involves the recruitment of MLL1 and MLL2 H3K4 histone methyltransferase complexes. Oncogene.

[36] Kuo H-P, Wang Z, Lee D-F, Iwasaki M, Duque-Afonso J, Wong SH, Lin C-H, Figueroa ME, Su J, Lemischka IR (2013). Epigenetic roles of MLL oncoproteins are dependent on NF-κB. Cancer Cell.

