# Supplementary figures and images for: Thrombin-induced, TNFR-dependent miR-181c downregulation promotes MLL1 and NF-κB target gene expression in human microglia
Source: J Neuroinflammation. 2017 Jun 29;14:132. doi: 10.1186/s12974-017-0887-5 (PMC5492717; doi:10.1186/s12974-017-0887-5)

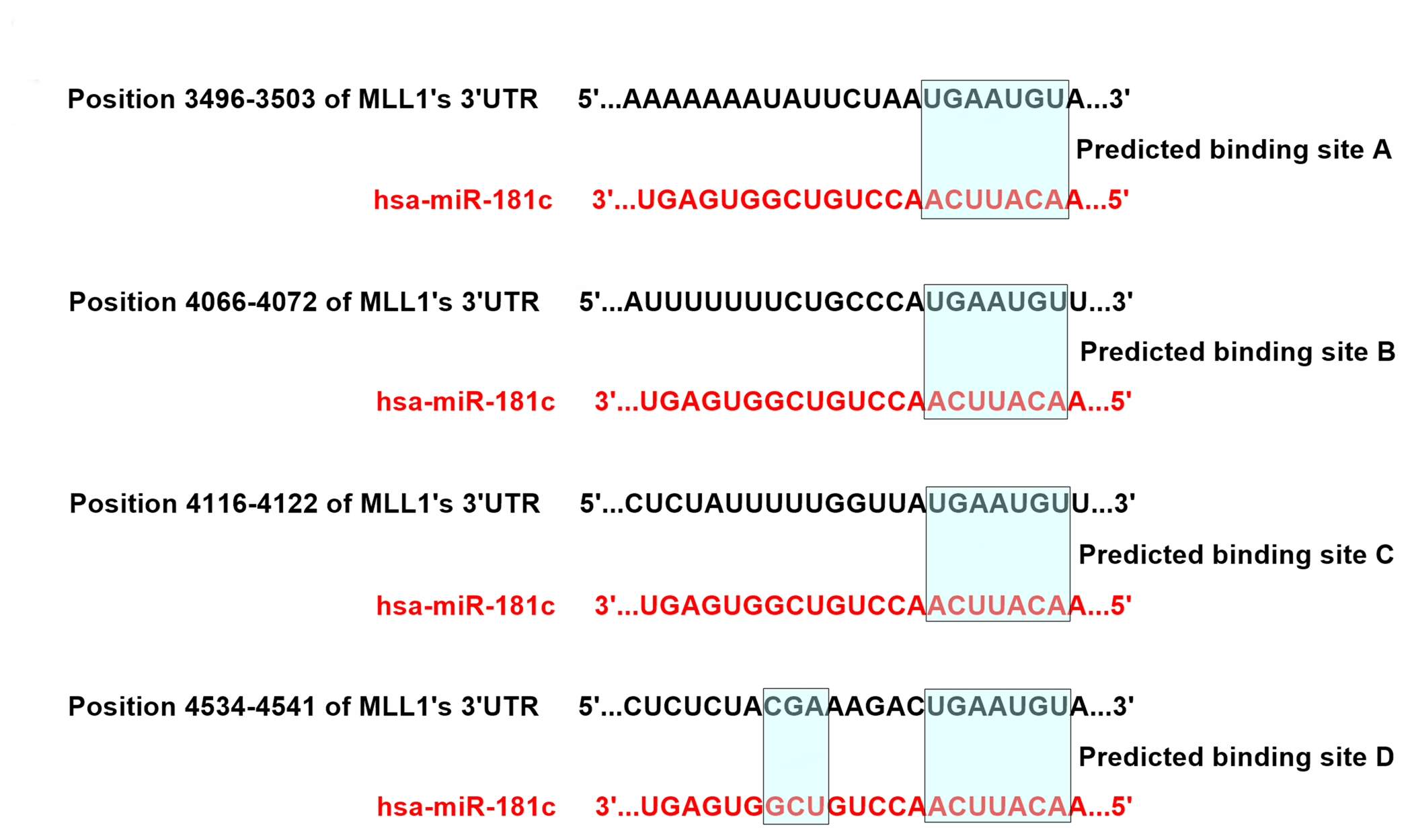

Supplement: Supplementary file 1 — miR-181c’s putative binding sites on MLL1’s 3′-UTR. Four putative binding sites for miR-181c on the 3′-UTR of MLL1 based on the TargetScan search. (TIF 429 kb) [file 12974_2017_887_MOESM1_ESM.tif]

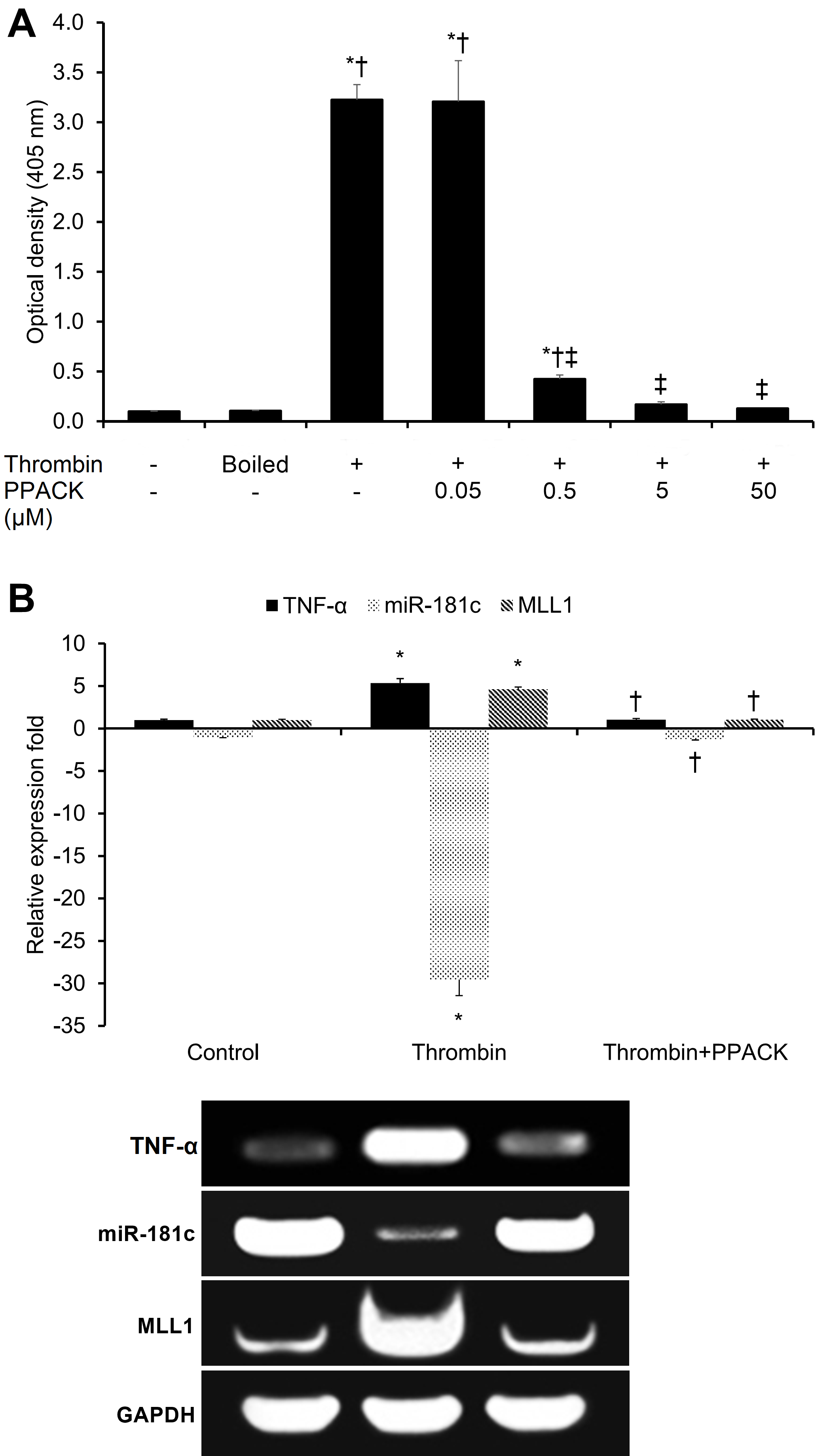

Supplement: Supplementary file 2 — Thrombin’s proteolytic activity contributed to its effects upon miR-181c and MLL1 expression in human microglia. (A) Validation of the thrombin-specific proteolytic inhibitor PPACK’s inhibition of thrombin activity. Thrombin’s proteolytic activity was measured via a chromogenic assay following pre-incubation in the absence or presence of various concentrations of PPACK. Heat-inactivated (boiled) thrombin was applied as a negative control. *p < 0.05 versus control, †p < 0.05 versus boiled thrombin, ‡p < 0.05 versus thrombin. (B) Pre-incubating with PPACK significantly inhibited thrombin’s effects upon miR-181c and MLL1 expression. *p < 0.05 versus control, †p < 0.05 versus thrombin. (TIF 598 kb) [file 12974_2017_887_MOESM2_ESM.tif]

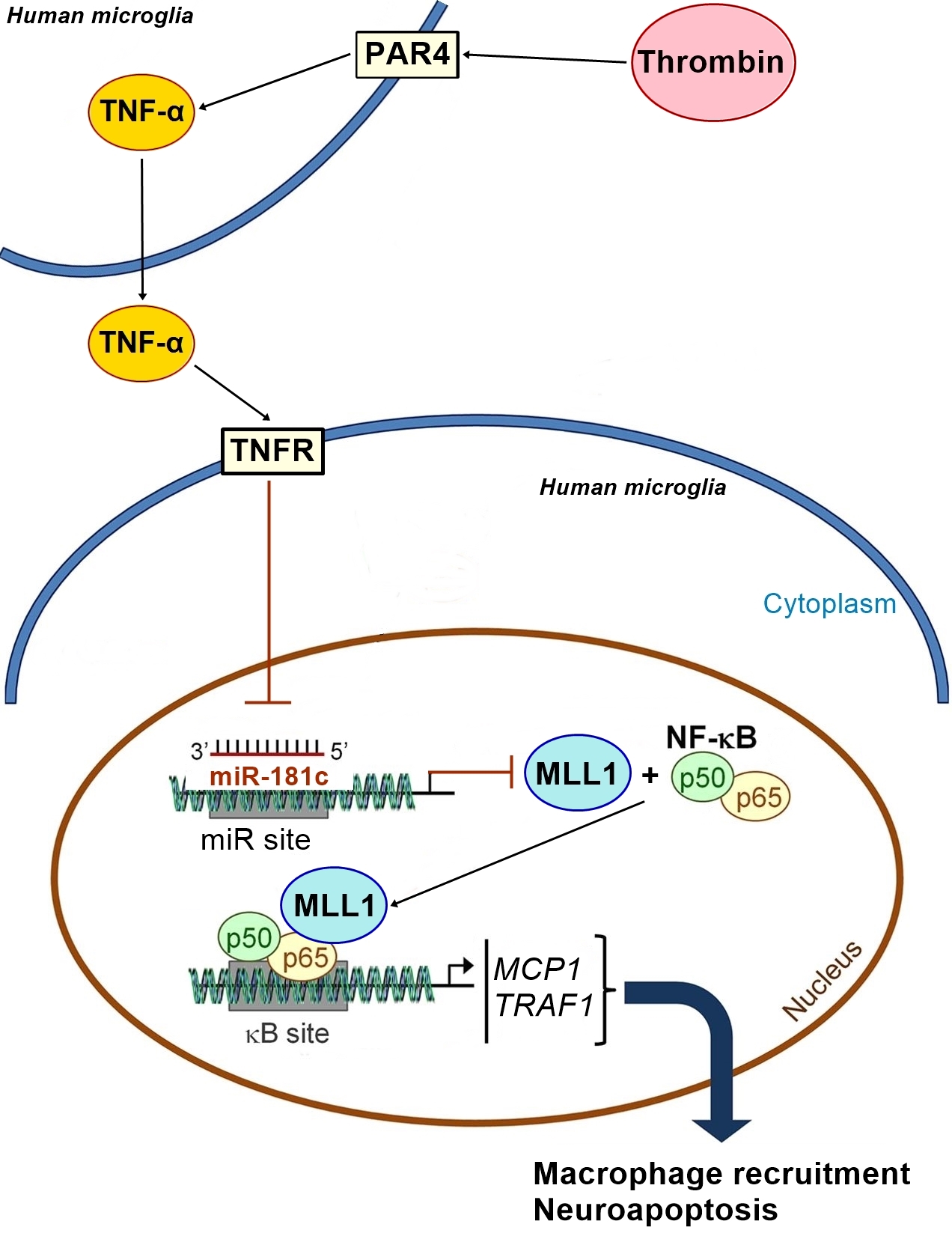

Supplement: Supplementary file 3 — Schematic overview of thrombin’s effects upon miR-181c and MLL1 in human microglia. Thrombin (via PAR4) induces TNF-α secretion from human microglia [21]. Thrombin-induced TNF-α (via TNFR) suppresses miR-181c levels. This suppression of the inhibitory miR-181c promotes MLL1 expression, increases NF-κB activity, and upregulates downstream NF-κB target gene expression in human microglia. (JPG 456 kb) [file 12974_2017_887_MOESM3_ESM.jpg]
